# Supplementary material for: Tracking the financial flows of Indonesia’s COVID-19 vaccination program
Source: PLOS Glob Public Health. 2025 Aug 5;5(8):e0005041. doi: 10.1371/journal.pgph.0005041 (PMC12324125; doi:10.1371/journal.pgph.0005041)
Supplement: S1 Appendix — (DOCX) [file pgph.0005041.s001.docx]

**S1 Appendix. Operational Definition of Variables for Cost Classification**

| **Variable** | **Operational Definition** | **Category** | **Definition** |
| --- | --- | --- | --- |
| Financial Sources (FS) | The financial source dimension provides an overview of the units/institutions/sources that provide health costs | National Budget and Expenditure (APBN - *Anggaran Pendapatan dan Belanja Negara*) | Funding for the implementation of vaccination program activities comes from the national budget (Deconcentration, Specific Allocation Fund (DAK - *Dana Alokasi Khusus*) Non-Physical, or Health Operational Assistance (BOK - Bantuan Operasional Kesehatan)). This category was chosen if it was not explained in detail from the results of the secondary data collection on financing what specific type of APBN was used. |
|  |  | Regional Budget and Expenditure (APBD - *Anggaran Pendapatan dan Belanja Daerah*) | Funding for the implementation of vaccination program activities comes from the Regional Budget and Expenditure (APBD). This category was chosen if it was not explained in detail from the results of the secondary data collection what  specific type of APBD was used. |
|  |  | National Budget and Expenditure -  Non-Physical Specific Allocation Fund (APBN - BOK/ DAK Non Fisik) | Health Operational Fund (BOK), is a Non-Physical Specific Allocation Fund (DAK) sourced from the National Budget which is allocated to regional governments for operational expenditure of national priority programmes for health offices and primary health care as executors of health programmes. |
|  |  | National Budget and Expenditure - Regional Incentive Funds (APBN - DID) | Regional Incentive Funds (DID - *Dana Insentif Daerah*) are funds sourced from the National Budget that are given to certain regions based on certain criteria aime to rewarding improvement and/or achievement of certain performance in the field of regional financial governance. |
|  |  | Regional Budget and Expenditure - Unexpected Expenditure Funds (APBD - BTT) | Unexpected Expenditures Funds (BTT - *Belanja Tidak Terduga*) are funds sourced from the Regional Budget and refunds of overpayments for previous years' regional revenues that are used to budget for emergency expenditures including urgent needs and social assistance that cannot be predicted and planned in advance . |
|  |  | Regional Budget and Expenditure - Refocusing of General Allocation Fund/ Revenue Sharing Fund (APBD - Refocusing DAU/DBH) | Provincial and Regency / City Governments provide funding support in the Regional Budget for the COVID-19 vaccination program in their respective regions whose funds can be sourced from the General Allocation Fund (DAU - *Dana Alokasi Umum*) and Revenue Sharing Fund (DBH - *Dana Bagi Hasil*). |
| Health Care (HC) | The health care dimension covers all the activities that aim to improve, enhance and maintain health status and minimize the risk of illness | Supporting Services | Encompass activities such as advocacy, meeting refreshments, and speaker honoraria |
|  |  | Medical Equipment / Materials | Cover pharmaceuticals, disposable medical supplies, and other medical devices |
|  |  | Public Health and Prevention Services | Costs associated with program implementation, such as meals during vaccination activities, travel expenses, and honoraria for vaccination teams |
|  |  | Other Health Services | This category includes community empowerment and other health services that are not defined specifically |
| Program (PR) | The program dimension captures whether expenditures are specific to a certain health program or integrated with other health programs. | Specific | This Specific category is for budget allocations that are specifically used for the COVID-19 vaccination program only, not combined with other programs. |
|  |  | Integrated | This Integrated category is for budget allocations that are not only used for the COVID-19 vaccination program, but are combined with other programs. |
| Health Inputs (HI) | The health inputs dimension is the type of input purchased by service/program providers to carry out activities | Investments | This category includes procurement for goods and services that have a longer use value such as land/building purchases, non-medical equipment procurement, medical equipment procurement, vehicle procurement, scholarships for employees, etc. |
|  |  | Operational | This category includes personnel expenditure, remuneration of independent professionals, expenditure on materials and services (medicines, medical consumables, non-medical equipment, stationery, travel, accommodation, utilities, other services, etc., etc.). |
|  |  | Maintenance | This category includes maintenance of land, buildings, medical and non-medical equipment, vehicles, other maintenance, and personnel/employee training. |

(Source: RI – USAID, 2021)[^37^](https://www.zotero.org/google-docs/?keFqbP)
