# Supplementary material for: Tracking the financial flows of Indonesia’s COVID-19 vaccination program
Source: PLOS Glob Public Health. 2025 Aug 5;5(8):e0005041. doi: 10.1371/journal.pgph.0005041 (PMC12324125; doi:10.1371/journal.pgph.0005041)
Supplement: S2 Appendix — (DOCX) [file pgph.0005041.s002.docx]

**S2 Appendix. Data Collection Matrix**

| **Province** | **Data Source** | **Quantitative Process** | **Qualitative Process** | **Incompleteness / Issues** |
| --- | --- | --- | --- | --- |
| Bali | COVID-19 vaccine expenditure data submitted by Health Offices using provided reporting format and supporting document | Compilation and validation of submitted expenditure data | In-Depth Interviews (IDI) with PHO’s staff | Complete |
| Central Sulawesi | COVID-19 vaccine expenditure data submitted by Health Offices using provided reporting format and supporting document | Compilation and validation of submitted expenditure data | In-Depth Interviews (IDI) with PHO’s staff | Complete |
| Papua | COVID-19 vaccine expenditure data submitted by Health Offices using provided reporting format and supporting document | Compilation and validation of submitted expenditure data | In-Depth Interviews (IDI) with PHO’s staff | Expenditure data provided as a total amount without breakdown by component, limiting comparison of detailed expenditures. |
| Jakarta | COVID-19 vaccine expenditure data submitted by Health Offices using provided reporting format and supporting document | Compilation and validation of submitted expenditure data | In-Depth Interviews (IDI) with PHO’s staff | Jakarta Province has a different administrative system, with no PHO expenditures for COVID-19 vaccination activities and all budget realization data managed separately by district health offices |
| Maluku | COVID-19 vaccine expenditure data submitted by Health Offices using provided reporting format and supporting document | Compilation and validation of submitted expenditure data | In-Depth Interviews (IDI) with PHO’s staff | Complete |
| Lampung | COVID-19 vaccine expenditure data submitted by Health Offices using provided reporting format and supporting document | Compilation and validation of submitted expenditure data | In-Depth Interviews (IDI) with PHO’s staff | Complete |
