# Supplementary material for: Tracking the financial flows of Indonesia’s COVID-19 vaccination program
Source: PLOS Glob Public Health. 2025 Aug 5;5(8):e0005041. doi: 10.1371/journal.pgph.0005041 (PMC12324125; doi:10.1371/journal.pgph.0005041)
Supplement: S3 Appendix — (DOCX) [file pgph.0005041.s003.docx]

**S3 Appendix. In-Depth Interview Participant**

| **No** | **In-Depth Interview Participant** |
| --- | --- |
| 1 | Lampung Provincial Health Office |
| 2 | Bali Provincial Health Office |
| 3 | Central Sulawesi Provincial Health Office |
| 4 | Maluku Provincial Health Office |
| 5 | Tanggamus District Health Office |
| 6 | Gianyar District Health Office |
| 7 | Sigi District Health Office |
| 8 | Seram Bagian Barat District Health Office |
