# Supplementary material for: Tracking the financial flows of Indonesia’s COVID-19 vaccination program
Source: PLOS Glob Public Health. 2025 Aug 5;5(8):e0005041. doi: 10.1371/journal.pgph.0005041 (PMC12324125; doi:10.1371/journal.pgph.0005041)
Supplement: S4 Appendix — (DOCX) [file pgph.0005041.s004.docx]

**S4 Appendix. Focus Group Discussion Participant**

| **No** | **Ministries** |
| --- | --- |
| 1 | Directorate General of Population and Civil Registration, Ministry of Home Affairs |
| 2 | Directorate General of Pharmaceuticals and Medical Devices, Ministry of Health |
| 3 | Directorate of Pharmaceutical Management and Services, Ministry of Health |
| 4 | Directorate of Natural Resources and Environmental Utilization, Ministry of Village, Development of Disadvantaged Regions and Transmigration |
| 5 | Directorate General of Disease Prevention and Control, Ministry of Health |
| 6 | Directorate General for Acceleration of Development of Disadvantaged Regions, Ministry of Village, Development of Disadvantaged Regions and Transmigration |
| **No** | **Subnational Government Agencies** |
| 1 | Bali Provincial Population and Civil Registration Office |
| 2 | Lampung Provincial Population and Civil Registration Office |
| 3 | Bali Provincial Health Office |
| 4 | Maluku Provincial Health Office |
| 5 | Central Sulawesi Provincial Health Office |
| 6 | Lampung Provincial Health Office |
| 7 | Seram Bagian Barat District Health Office |
| 8 | Sigi District Health Office |
| 9 | Gianyar District Health Office |
| 10 | Tanggamus District Health Office |
| 11 | Community health center (puskesmas), Gianyar |
| 12 | Community health center (puskesmas), Tanggamus |
| **No** | **Civil Society Organizations** |
| 1 | Civil Society Coalition on Vaccine Access for Indigenous Peoples and Vulnerable Groups |
| 2 | Empowerment of Female Heads of Households (PEKKA) |
| 3 | The Indonesian Association of Women with Disabilities - Jakarta’s Headquarter |
| 4 | The Indonesian Association of Women with Disabilities - Bali |
| 5 | Filantropi Indonesia |
| 6 | Public Interest Research and Advocacy Center (PIRAC) |
