# Supplementary material for: Tracking the financial flows of Indonesia’s COVID-19 vaccination program
Source: PLOS Glob Public Health. 2025 Aug 5;5(8):e0005041. doi: 10.1371/journal.pgph.0005041 (PMC12324125; doi:10.1371/journal.pgph.0005041)
Supplement: S5 Appendix — (DOCX) [file pgph.0005041.s005.docx]

**S5 Appendix. Budget and Expenditure Trends for the COVID-19 Vaccination Program**

|  | | **Health Office** | **Budget (US$)** | **Expenditure (US$)** | **% Realization** |
| --- | --- | --- | --- | --- | --- |
| **Year** | **2021** | Bali PHO | 517.478 | 53.445 | 10 |
|  |  | Central Sulawesi PHO | 71.52 | 54.548 | 76 |
|  |  | Lampung PHO | 677.827 | 677.827 | 100 |
|  |  | Maluku PHO | 21.154 | 7.469 | 35 |
|  |  | Gianyar DHO | 1.566 | 818 | 52 |
|  |  | Sigi DHO | 449.726 | 382.377 | 85 |
|  |  | Tanggamus DHO | 24.462 | 24.462 | 100 |
|  |  | Seram Bagian Barat DHO | 4.962 | 4.962 | 100 |
|  | **2022** | Bali PHO | 3.727 | 2.689 | 72 |
|  |  | Central Sulawesi PHO | 118.815 | 101.719 | 86 |
|  |  | Lampung PHO | 19.627 | 19.627 | 100 |
|  |  | Maluku PHO | 178.182 | 56.425 | 32 |
|  |  | Gianyar DHO | 409 | 367 | 90 |
|  |  | Sigi DHO | 198.526 | 198.436 | 100 |
|  |  | Tanggamus DHO | 354.664 | 316.024 | 89 |
|  |  | Seram Bagian Barat DHO | 20.25 | 19.545 | 97 |
