# Supplementary material for: Tracking the financial flows of Indonesia’s COVID-19 vaccination program
Source: PLOS Glob Public Health. 2025 Aug 5;5(8):e0005041. doi: 10.1371/journal.pgph.0005041 (PMC12324125; doi:10.1371/journal.pgph.0005041)
Supplement: S6 Appendix — (DOCX) [file pgph.0005041.s006.docx]

**S6 Appendix. Expenditure per capita (US$) and vaccine coverage**

| **Health Office** | **Total Expenditure (2021-2022)** | **Vaccine Targets** | **Expenditure per Capita [Total Expenditure/Vaccine Targets]** | **Vaccine Coverage (%)** |
| --- | --- | --- | --- | --- |
| Bali PHO | US$56,134 | 3774174 | 0.015 | 97.94 |
| Central Sulawesi PHO | US$156,267 | 2135907 | 0.073 | 53.95 |
| Lampung PHO | US$697,454 | 7558816 | 0.092 | 66.91 |
| Maluku PHO | US$63,894 | 1613247 | 0.040 | 45 |
| Gianyar DHO | US$1,185 | 452830 | 0.003 | 90.4 |
| Sigi DHO | US$580,813 | 184952 | 3.140 | 37.1 |
| Tanggamus DHO | US$102,312 | 536701 | 0.191 | 60.2 |
| Seram Bagian Barat DHO | US$24,507 | 183820 | 0.133 | 24.5 |
