# Supplementary material for: Tracking the financial flows of Indonesia’s COVID-19 vaccination program
Source: PLOS Glob Public Health. 2025 Aug 5;5(8):e0005041. doi: 10.1371/journal.pgph.0005041 (PMC12324125; doi:10.1371/journal.pgph.0005041)
Supplement: S7 Appendix — (DOCX) [file pgph.0005041.s007.docx]

**S7 Appendix. Expenditure Tracking**

|  | **Health Office** | **Financial Source** | | | | | **Health Care Function** | | | | **Program** | | **Health Input** | | |
| --- | --- | --- | --- | --- | --- | --- | --- | --- | --- | --- | --- | --- | --- | --- | --- |
|  |  | **APBD-BTT** | **APBD - Refocusing DAU/DBH** | **APBD** | **APBN - DID** | **APBN - BOK/DAK Non Fisik** | **Medical Equipment/ Materials** | **Public Health and Prevention Services** | **Supporting Service** | **Other Health Services** | **Specific** | **Integrated** | **Operational** | **Maintenance** | **Investment** |
| 2021 | Bali PHO |  | 53,445 |  |  |  |  | 38,166 | 15,279 |  | 47,108 | 6,337 | 45,872 | 508 | 7,065 |
|  | Central Sulawesi PHO |  |  | 54,548 |  |  |  | 41,958 | 12,590 |  | 54,548 |  | 54,548 |  |  |
|  | Lampung PHO |  |  | 677,827 |  |  | 675,567 |  |  | 2,260 | 62,245 | 615,582 | 143,847 |  | 532,911 |
|  | Maluku PHO | 7,469 |  |  |  |  | 7,469 |  |  |  | 7,469 |  | 7,469 |  |  |
|  | Gianyar DHO |  |  |  |  | 818 |  |  | 818 |  |  | 818 |  | 818 |  |
|  | Sigi DHO |  | 382,377 |  |  |  | 80,315 | 269,101 | 32,961 |  | 290,212 | 92,164 | 286,156 | 32,961 | 63,260 |
|  | Tanggamus DHO |  |  | 24,462 |  |  |  | 24,462 |  |  | 24,462 |  | 24,462 |  |  |
|  | Seram Bagian Barat DHO |  |  |  |  | 4,962 | 4,962 |  |  |  | 4,962 |  | 4,962 |  |  |
| 2022 | Bali PHO | 2,689 |  |  |  |  |  | 2,689 |  |  | 2,689 |  | 2,689 |  |  |
|  | Central Sulawesi PHO |  |  | 101.719 |  |  |  | 86,166 | 15,552 |  | 65,819 | 35,899 | 101,719 |  |  |
|  | Lampung PHO |  |  | 11,627 |  |  | 10,192 | 9,435 |  |  | 9,435 | 10,192 | 19,627 |  |  |
|  | Maluku PHO | 56,425 |  |  |  |  | 28,469 |  | 27,956 |  | 56,425 |  | 28,469 | 27,956 |  |
|  | Gianyar DHO |  |  |  |  | 367 |  |  | 367 |  |  | 367 |  | 367 |  |
|  | Sigi DHO |  | 102,874 |  | 95,562 |  |  | 198,435 |  |  | 179,481 | 18,954 | 198,435 |  |  |
|  | Tanggamus DHO |  |  | 77,850 |  | 238,174 |  | 316,024 |  |  | 316,024 |  | 316,024 |  |  |
|  | Seram Bagian Barat DHO |  |  |  | 7,847 | 11,698 | 4,522 | 3,325 | 11,698 |  | 19,545 |  | 7,847 | 11,698 |  |
