# Supplementary material for: Tracking the financial flows of Indonesia’s COVID-19 vaccination program
Source: PLOS Glob Public Health. 2025 Aug 5;5(8):e0005041. doi: 10.1371/journal.pgph.0005041 (PMC12324125; doi:10.1371/journal.pgph.0005041)
Supplement: Attachment — Submitted filename: Review PLOS GPH _ vaccine COVID.docx [file pgph.0005041.s008.docx]

1. Introduction: Please specify the number of doses required for a complete vaccination in Indonesia. Additionally, clarify whether the 23% coverage in Papua refers to the primary dose or the complete vaccine series. Also, provide a definition of the vulnerable groups mentioned.
2. Methods: There is no explanation of how the authors selected the districts. It appears that Sigi had lower coverage than Central Sulawesi—please clarify this.
3. Methods: For better flow, consider moving the Study Population section above the explanation of the Quantitative and Qualitative Design.
4. Methods: What was the purpose of the qualitative study? Was it used for triangulation? If so, what aspects were being triangulated? Did it aim to explore challenges related to vaccination?
5. Figure 2: I suggest removing this figure and incorporating its findings into the text instead.
6. Figure 3: In 2021, Lampung spent $677,827, which was significantly higher than any other province. Given that Lampung’s GDP was only twice that of Bali, how did Lampung manage such high expenditures? Were there additional financial resources involved?
7. Figures 10–11: These figures illustrate differences in cost allocations between provinces and districts, and the variations are striking. For example, Lampung and Maluku allocated most of their budget to equipment, while Bali and Sulawesi prioritized preventive services. Could it be that similar expenses were classified under different cost categories in the NHA/PHA? The same pattern is observed at the district level. Additionally, please provide more details about the cost categories to improve reader understanding—for instance, where were travel costs included?
8. Number of figures: There are many figures in the manuscript. Please consider merging some into a single figure with multiple panels.
9. Spending on routine vaccines: Did the authors analyze spending on routine vaccines at the provincial and district levels? This analysis could help explain why certain provinces invested more than others and had higher vaccine coverage. It is reasonable to assume that provinces or districts with higher routine vaccine coverage and spending would also have higher COVID-19 vaccine coverage. In the case of Lampung, which invested more in the cold chain, was this due to an already well-established routine vaccination infrastructure?
10. Funding allocation: Some regions allocated more funding to “Support” than to the “Core Component.” Can the authors provide an explanation for this finding? Were the vaccines donated by entities other than the local government?
11. Lines 427–431: Was the spending correlated with the COVID-19 burden in each region? There is no explanation provided. It is important to assess how the system responded to the burden of the pandemic.!
