# Supplementary material for: Tracking the financial flows of Indonesia’s COVID-19 vaccination program
Source: PLOS Glob Public Health. 2025 Aug 5;5(8):e0005041. doi: 10.1371/journal.pgph.0005041 (PMC12324125; doi:10.1371/journal.pgph.0005041)
Supplement: Attachment — Submitted filename: Response to Reviewers.docx [file pgph.0005041.s010.docx]

**Response to Reviewer**

Dear Editor,

We appreciate you and the reviewers for your precious time in reviewing our paper and providing valuable comments. It was your valuable and insightful comments that led to possible improvements in the current version. The authors have carefully considered the comments and tried our best to address every one of them. We hope the manuscript after careful revisions meet your high standards. The authors welcome further constructive comments if any.

Below we provide the point-by-point responses. All revisions can be found in the tracked changes version as well as in the clean version of the manuscript.

Sincerely,

Ayudina Larasanti

Senior Public Health Research Officer

**Center for Indonesia's Strategic Development Initiatives (CISDI)**

Jl. Probolinggo No. 40C, Menteng, Jakarta Pusat 10350

**Comments to the Author**

1. Does this manuscript meet PLOS Global Public Health’s publication criteria? Is the manuscript technically sound, and do the data support the conclusions? The manuscript must describe methodologically and ethically rigorous research with conclusions that are appropriately drawn based on the data presented.

Reviewer #1: No

Reviewer #2: Yes

Reviewer #3: Partly

Reviewer #4: Yes

2. Has the statistical analysis been performed appropriately and rigorously?

Reviewer #1: N/A

Reviewer #2: Yes

Reviewer #3: No

Reviewer #4: N/A

3. Have the authors made all data underlying the findings in their manuscript fully available (please refer to the Data Availability Statement at the start of the manuscript PDF file)?

The PLOS Data policy requires authors to make all data underlying the findings described in their manuscript fully available without restriction, with rare exception. The data should be provided as part of the manuscript or its supporting information, or deposited to a public repository. For example, in addition to summary statistics, the data points behind means, medians and variance measures should be available. If there are restrictions on publicly sharing data—e.g. participant privacy or use of data from a third party—those must be specified.

Reviewer #1: Yes

Reviewer #2: Yes

Reviewer #3: No

Reviewer #4: No

4. Is the manuscript presented in an intelligible fashion and written in standard English?

PLOS Global Public Health does not copy its accepted manuscripts, so the language in submitted articles must be clear, correct, and unambiguous. Any typographical or grammatical errors should be corrected at revision, so please note any specific errors here.

Reviewer #1: No

Reviewer #2: Yes

Reviewer #3: Yes

Reviewer #4: Yes

**Comments from Reviewer 1**

|  | **Reviewer Comment** | **Response** | **Changes Made in Manuscript** |
| --- | --- | --- | --- |
| 1 | Lines 45-47, when you refer to coverage is it one dose, two doses? Please explain. | Thank you for your question. The vaccination coverage data referenced in Lines 45–47 are from the WHO COVID-19 dashboard, which reports the percentage of the population that has received **at least one dose** of COVID-19 vaccine. This includes individuals who have received only one dose as well as those who have completed the primary series or received booster doses. We have revised the text to clarify this point. | Lines 48 to 51:  *As of mid-2023, the proportion of the population that had received at least one dose of COVID-19 vaccine, reached 67.73% in lower-middle-income countries (LMICs) and 33.8% in low-income countries (LICs), markedly lower than the 80.27% and 80.77% observed in high-income and upper-middle-income countries (UMICs) respectively* |
| 2 | Line 79, there are many more studies that could be cited, e.g. <https://doi.org/10.1016/j.vhri.2022.04.004>. | Thank you for the helpful suggestion. We have reviewed the recommended study (Morales-Zamora et al, 2022) and found it relevant to our topic. We have now included this citation in manuscript, along with a few additional studies to strengthen the contextual background. | Lines 91 to 96:  *For example, a study in Colombia comparing two COVID-19 vaccination strategies found that prioritizing high-risk groups was consistently more cost-effective in low-resource settings, while a study in South Africa’s West Rand district, which estimated delivery costs across multiple channels, identified vaccine procurement and staff time as key cost drivers, highlighting the importance of detailed expenditure tracking to understand vaccination program finances.* |
| 3 | Overall, the description does not have an orderly structure and is also very simplistic. Reviewer 1 doesn't think the study adds value to the literature | Although our study appears quite simplistic, it involved an extensive process encompassing data collection, processing, and displaying the graphs. We employed expenditure data rather than budget. As a result, in this article we provided real expenses that have been confirmed to program coordinators at every region through an interview. This activity was part of the analysis in order to perform micro-costing so that we were able to calculate personnel, supplies, equipment, training and more. Additionally, we classified this financial flow into "core" and "support" to explore whether the money is spent mostly to directly to the beneficiaries or to support activities such as meeting or coordination tasks. To our best knowledge, our findings are important as insights for improving health management systems where one region achieves its coverage, while the other falls short, particularly in local context. | - |
| 4 | Revise the English wording of the paper | Thank you for your valuable feedback. We have carefully revised the English wording throughout the manuscript to improve clarity, grammar, and overall readability. |  |

**Comments from Reviewer 2**

|  | **Reviewer Comment** | **Response** | **Changes Made in Manuscript** |
| --- | --- | --- | --- |
| 1 | Introduction:   a. Please specify the number of doses required for a complete vaccination in Indonesia. b. Clarify whether the 23% coverage in Papua refers to the primary dose or the complete vaccine series. c. Provide a definition of the vulnerable groups mentioned. | Thank you for your comment. We have added information to the manuscript to clarify this point.   a. In Indonesia, the term "complete vaccination" refers to the completion of the primary vaccination series. According to the Technical Guidelines for the Implementation of Vaccination (HK.01.07/MENKES/4638/2021), the primary vaccination consists of two doses. b. The 23.38% coverage in Papua refers to the percentage of the population that has completed the primary vaccination series. c. The definition of vulnerable groups, as outlined in our previous study [(Herlinda, 2023)](https://cdn.cisdi.org/reseach-document/fnm-EnglishFull-Report-Policy-Study-A-Study-on-The-Inclusivity-of-The-COVID-19-Vaccination-Program-copypdf-1715654239536-fnm.pdf), and aligned with frameworks from the United Nations (UN) and the World Health Organization (WHO), refers to individuals who face significant barriers that increase their risk of COVID-19 infection and/or the severity of the disease. These barriers include systemic marginalization or discrimination, low health literacy, poor initial health status, limited access to public healthcare services, and other factors that contribute to their vulnerability. | Lines 57 to 60:  *As outlined in the Technical Guidelines for the Implementation of Vaccination (Regulation No. HK.01.07/MENKES/4638/2021), in the context of Indonesia, the term “complete vaccination” refers to the completion of the primary vaccination series, which generally consists of two doses for most COVID-19 vaccines.*  Lines 70 to 74:  *In this study, we define vulnerable groups as individuals who face significant barriers that increase their risk of COVID-19 infection and/or the severity of the disease. These barriers include systemic marginalization or discrimination, low health literacy, poor initial health status, limited access to public healthcare services, and other factors that contribute to their vulnerability* |
| 2 | Methods: There is no explanation of how the authors selected the districts. It appears that Sigi had lower coverage than Central Sulawesi—please clarify this. | Thank you for this insightful comment. We appreciate the opportunity to clarify our district selection process.  As described in the revised Methods section, we first purposively selected provinces to represent three categories of primary dose vaccination coverage in 2023—over 70%, 50–70%, and under 50%. Within each selected province, one district was then **purposively selected** to capture intra-provincial variation and obtain diverse insights from District Health Offices (DHOs).  Sigi District, located within Central Sulawesi Province, was selected to represent the low-coverage category. It was chosen not only based on its vaccination performance, but also because of its demographic and geographic challenges, including rural and remote areas with limited access to healthcare services. These characteristics offered valuable perspectives on structural barriers affecting vaccine delivery and financing. Other districts in our sample also reflect diverse contexts. For example, Seram Bagian Barat and parts of Tanggamus experience similar accessibility constraints due to island and mountainous terrain, respectively, while Gianyar represents a more urbanized and better-connected area. This variation was intended to enrich our comparative analysis of COVID-19 vaccine financial flows across different regional settings. We have revised the Methods section to reflect these clarifications. | Lines 126 to 132:  *Within each selected province, one district was purposively chosen to capture intra-provincial variation and gather diverse perspectives from District Health Offices (DHOs). District selection considered not only vaccination coverage but also demographic and geographic characteristics. For instance, Sigi, Seram Bagian Barat, and parts of Tanggamus face accessibility challenges due to remote and mountainous terrains, while Gianyar represents a more urbanized district with stronger healthcare infrastructure. These varied contexts provided a solid foundation for comparing COVID-19 vaccine financial flows across regions.* |
| 3 | Methods: For better flow, consider moving the Study Population section above the explanation of the Quantitative and Qualitative Design. | Thank you for your helpful suggestion. We agree that moving the "Study Population" section above the "Study Design" will improve the flow of the manuscript. We have made the revision accordingly. | The study population section has been moved before the study design section |
| 4 | Methods: What was the purpose of the qualitative study? Was it used for triangulation? If so, what aspects were being triangulated? Did it aim to explore challenges related to vaccination? | Thank you for your comment. The qualitative study of our study was indeed designed for triangulation purposes, as well as for gaining deeper insights into the financing and implementation challenges of the COVID-19 vaccination program.  As stated in our manuscript, the FGDs and in-depth interviews were conducted to **triangulate and contextualize data.** Specifically aimed to triangulate findings from our quantitative analysis with firsthand experiences and perceptions of key stakeholders involved in the program. This helped us validate and enrich our understanding of **budgeting processes, resource allocation, and coordination across health system levels***.*. In addition, the qualitative study sought to explore challenges related to the planning and financing of the COVID-19 vaccination program. These included **operational bottlenecks, inter-agency coordination issues, and local-level implementation constraints**, particularly those affecting fund allocation and usage.  We have clarified this purpose in the revised manuscript to ensure it is more explicitly stated. | Lines 181 to 184:  *The qualitative study aimed to triangulate and contextualize findings from the quantitative data, and explore implementation challenges in the COVID-19 vaccination program, particularly related to financing and fund management. Triangulation focused on validating budgeting processes, resource allocation, and coordination across levels.* |
| 5 | Figure 2: Reviewer 2 suggest removing this figure and incorporating its findings into the text instead. | Thank you for your suggestion. We agree that the information presented in Figure 2 can be effectively conveyed through the narrative. Accordingly, we have removed Figure 2 and incorporated its key findings into the main text to improve clarity and streamline the overall structure of the manuscript. | Lines 214 to 220:  *During the COVID-19 response from 2020 to 2022, the budget allocation for the vaccination program reflected the shifting priorities and intensity of the pandemic. In 2020, US$2.497 million was allocated, primarily for vaccine procurement. The highest allocation occurred in 2021, reaching US$5.171 million, driven by the national vaccine rollout and peak infection and mortality rates. By 2022, the allocation decreased significantly to US$1.108 million, reflecting reduced overall COVID-19 funding amid more controlled transmission and declining public demand for vaccination.* |
| 6 | Figure 3: In 2021, Lampung spent $677,827, which was significantly higher than any other province. Given that Lampung's GDP was only twice that of Bali, how did Lampung manage such high expenditures? Were there additional financial resources involved? | In 2021, provinces had flexibility in using national (APBN) and regional (APBD) budgets for COVID-19 vaccination, resulting in varied funding amounts and sources influenced by fiscal capacity, government commitment, institutional strength, access barriers, and private sector involvement. The central government did not set strict spending standards but required coordination to avoid duplicated costs.  Lampung’s high expenditure was driven by reallocations from the regional budget for medical equipment. Lampung needed cold room and cold chain infrastructure capable of maintaining temperatures below 8 degrees Celsius which were not included in the spending components of the other three provinces. This was due to inadequate prior facilities and the scale of vaccination in remote areas. | Lines 226 to 234:  *In 2021, Lampung recorded the highest expenditure among the four provinces, primarily due to reallocations and support from the regional budget for medical equipment. This was driven by the need to establish cold chain infrastructure capable of maintaining temperatures below 8 degrees Celsius, as the province lacked an adequate system prior to the pandemic and had to support a large-scale vaccination program in remote areas. Lampung’s larger population of 7,558,816 also contributed to its higher spending. In 2022, its expenditure declined sharply as major investments in medical equipment, cold chain, and infrastructure had already been made in the previous year.* |
| 7 | Figures 10–11: These figures illustrate differences in cost allocations between provinces and districts, and the variations are striking. For example, Lampung and Maluku allocated most of their budget to equipment, while Bali and Sulawesi prioritized preventive services.   a. Could it be that similar expenses were classified under different cost categories in the NHA/PHA? The same pattern is observed at the district level. b. Additionally, please provide more details about the cost categories to improve reader understanding—for instance, where were travel costs included? | a. We believe this possibility has been minimized. In this study, the classification of expenditures was conducted directly by the research team using the standardized NHA/PHA framework consistently across all provinces and districts. This approach ensured that similar expenditures were categorized uniformly, so the observed differences in allocation reflect actual spending priorities rather than inconsistencies in classification. b. Thank you for your suggestion. The operational definitions of the cost categories used in our classification are provided in Appendix 1. However, to enhance reader understanding, we have also revised the manuscript to include a brief explanation of the health care function (HC) categories as a footnote in the results section. | Lines 446 to 448:  *The health care function (HC) of the COVID-19 vaccination program comprises public health and prevention services, medical equipment and materials, supporting services, and other health services.*  Footnote  *Public health and prevention services include costs associated with program implementation, such as meals during vaccination activities, travel expenses, and honoraria for vaccination teams. Medical equipment and materials cover pharmaceuticals, disposable medical supplies, and other medical devices. Supporting services encompass activities such as advocacy, meeting refreshments, and speaker honoraria. Other health services involve community empowerment initiatives and additional health-related activities.* |
| 8 | Number of figures: There are many figures in the manuscript. Please consider merging some into a single figure with multiple panels. | Thank you for the suggestion. We have reviewed the figures in the manuscript and agree that some can be effectively combined. We have updated the figure numbering and corresponding references in the text accordingly | We have merged the following figures into multi-panel formats:   1. Figures 3 and 4 → **Figure 2 (a, b)** 2. Figures 5 and 6 → **Figure 3 (a, b)** 3. Figures 8 and 9 → **Figure 5 (a, b)** 4. Figures 10 and 11 → **Figure 6 (a, b)** 5. Figures 12 and 13 → **Figure 7 (a, b)** 6. Figures 14 and 15 → **Figure 8 (a, b)** |
| 9 | Spending on routine vaccines: Did the authors analyze spending on routine vaccines at the provincial and district levels? This analysis could help explain why certain provinces invested more than others and had higher vaccine coverage. It is reasonable to assume that provinces or districts with higher routine vaccine coverage and spending would also have higher COVID-19 vaccine coverage. In the case of Lampung, which invested more in the cold chain, was this due to an already well-established routine vaccination infrastructure? | Thank you for this valuable comment. We agree that analyzing provincial and district-level spending on routine vaccines would provide useful context for understanding variations in COVID-19 vaccine coverage and infrastructure readiness.  Unfortunately, we did not collect or analyze routine vaccine spending data at the subnational level since our focus mainly on COVID-19 vaccine expenditure. While we tried to find the data online, there is a lack of data publicly available, particularly disaggregated expenditure data like the ones we collected directly from the DHOs and PHOs.  We will revise the manuscript to:   - Clarify this limitation explicitly in the Methods and Discussion sections. - Acknowledge that provinces with stronger routine immunization systems and investments may have been better positioned to deliver COVID-19 vaccines, and that future research should explore these financial and infrastructural linkages.   Regarding Lampung, its high expenditure was driven by budget reallocations for medical equipment, particularly the need to establish cold rooms and cold chain infrastructure capable of maintaining temperatures below 8°C. We cautiously assume that its investment in the cold chain, while it may reflect its commitment to improve routine immunization infrastructure, may also indicate its existing immunization infrastructure was not adequate, hence the big investment. However, without specific routine vaccine expenditure data, we are unable to confirm this quantitatively. | Lines 111 to 114:  *The study focused on COVID-19 vaccine-related expenditure at the provincial and district levels; routine immunization spending data were not collected. Publicly available subnational data on routine immunization were sought but were unavailable and lacked sufficient detail to disaggregate different kinds of expenditures.*  Lines 632 to 637:  *The lack of publicly available, disaggregated financial data on routine immunization prevented examination of the potential relationship between past investments in routine immunization and COVID-19 vaccine delivery. Provinces with stronger routine immunization systems may have been better positioned to implement COVID-19 vaccination effectively. Future research should explore these financial and infrastructural linkages to better understand the role of existing immunization systems in pandemic response.* |
| 10 | Funding allocation: Some regions allocated more funding to "Support" than to the "Core Component."   a. Can the authors provide an explanation for this finding? b. Were the vaccines donated by entities other than the local government? | a. Thank you for the insightful question.   Several regions allocated more to supporting components than to core vaccination activities due to limited technical guidance, varying administrative capacities, and the perception that M&E or reporting activities were essential for accountability. In some cases, core elements such as vaccines or cold chain equipment were provided by the central government, prompting local governments to focus their budgets on complementary support. In regions with logistical challenges, higher spending on coordination or training was necessary to enable delivery. While these findings suggest high reliance on the national budget, this may also reinforce the importance of clearer national guidelines and regulation to ensure balanced and appropriate budget allocation across components.  “Core” means that funding goes to the beneficiaries directly, whereas “support” refers to activities that are supplemental to core functions. When beneficiaries receive vaccinations, for example, every dollar spent on the actual vaccination is considered “core”. Meetings and coordinations before the intervention, on the other hand, are classified as “support”. Basically, these two terms refer to the government regulation no 12 year 2019 and the regulation of ministry of home affairs of the republic of Indonesia no 64 year 2020. Mambea et al (2023) has translated such government regulations into “core” and “support” in terms of health financing in Indonesian context. In this paper, we refer to health financing in the Indonesian context.   b. Regarding vaccine supply, vaccines distributed to regions were provided by the central government through a structured, tiered system, starting from the Ministry of Health/ Biofarma, then to Provincial Health Offices (PHO), District Health Offices (DHO), and finally to vaccination sites such as puskesmas and clinics. In addition, private sector entities, state-owned enterprises (BUMN), and institutions such as the Indonesian National Armed Forces (TNI) also conducted vaccination initiatives in certain areas, using vaccines supplied through government channels or philanthropic organizations | Lines 565 to 570:  *These disproportionate allocations may stem from limited technical guidance and regulations, varying administrative capacities, and limited focus on accountability-related activities such as M&E. In some cases, core elements like vaccines or cold chain equipment were directly provided by the central government, leading subnational budgets to focus on complementary support. Additionally, logistical constraints in remote areas often required higher investment in coordination or training to enable delivery.*  Lines 575 to 578:  *“Core” refers to funding that goes directly to beneficiaries, such as vaccines distribution, cold chain equipment, and PPE. While “support” refers to activities that are supplemental to core functions, such as meetings and coordination before the intervention* |
| 11 | Lines 427–431: Was the spending correlated with the COVID-19 burden in each region? There is no explanation provided. It is important to assess how the system responded to the burden of the pandemic. | Thank you for raising this important point. Understanding the regional COVID-19 burden is indeed critical for assessing how spending aligned with pandemic needs. The cumulative burden, as defined by the CDC, includes infections, outpatient visits, hospitalizations, and deaths over time. However, we do not have sufficient data covering all these aspects, especially down to the district levels, which limits a comprehensive assessment of system responsiveness.  Within this limitation, we analyzed case fatality rate (CFR) trends (based on Indonesia Health Profile 2020-2023) as a partial indicator. After the 2021 vaccine rollout, CFRs generally declined in Central Sulawesi and Lampung, while Bali experienced a slight increase and Maluku showed fluctuations, likely due to very low case and death counts. However, by 2023, confirmed cases and deaths had dropped significantly across all provinces.  Although a direct correlation between spending and COVID-19 burden could not be established, these observations suggest that differences in fiscal capacity, government commitment, and access barriers played roles in shaping pandemic responses and vaccine financing allocation. | Lines 594 to 606:  *Understanding regional COVID-19 health burden, including infections, hospitalizations, and deaths, remains pivotal for effective allocation of resources and planning. However, the limited data available at the subnational level constrained a comprehensive assessment of system responsiveness. Within this constraint, case fatality rate (CFR) trends offer some insight. Following the 2021 vaccine rollout, CFRs over the 2020–2023 period generally declined in Central Sulawesi and Lampung, while Bali experienced a slight increase and Maluku showed fluctuations due to very low case and death numbers. However, confirmed cases and deaths dropped significantly across all provinces by 2023. While a direct correlation between spending and COVID-19 burden cannot be established, these findings provide preliminary insights into how variations in fiscal capacity, political commitment, national and regional priorities, access challenges, geography, existing immunization infrastructure, and private sector engagement influenced pandemic response and vaccine fund allocation.* |

**Comments from Reviewer 3**

|  | **Reviewer Comment** | **Response** | **Changes Made in Manuscript** |
| --- | --- | --- | --- |
| 1 | The manuscript provides an in-depth analysis of COVID-19 vaccine equity, health systems, and policy perspectives. The review of challenges and opportunities in vaccine distribution and access—particularly in Indonesia—is comprehensive, and the discussion on the economic evaluation of health interventions and the impact on health workers is timely. The extensive reference list demonstrates a robust engagement with current literature. | We sincerely thank the reviewer for the thoughtful and positive feedback. We greatly appreciate your recognition of the manuscript’s comprehensive analysis and timely contributions, particularly regarding vaccine equity, economic evaluation, and health workforce implications. Your comments affirm the relevance of our work. | - |
| 2 | However, the methodology section would benefit from additional detail regarding the costing processes and the interpretation of results. Although the Indonesia focus offers valuable context-specific insights, the generalizability of the findings to regions with differing health systems and socio-economic conditions is limited. Finally, further clarification is needed on how potential biases in data collection and analysis were addressed, and on the methods used to normalize the analysis, especially in light of the reliance on expert judgment due to data limitations. | We sincerely appreciate the reviewer’s insightful feedback. In response, we have revised the manuscript to address the points raised:   1. Expenditure data were collected across the selected provinces and districts using a specialised format developed by the research team by modified from the existing DHO format, to address the significant variation in financial reporting formats across regions, ensuring greater comparability in the data collected. Data were also gathered through in-depth interviews with key informants from health offices and a review of relevant program documentation to validate the expenditure information. Despite the provision of a structured format, challenges arose in comparing the quantitative data, as some health offices were unable to supply detailed expenditure information, even after the data had been validated through interviews(added in Appendix 2). To address this, we refined the microcosting analysis by resolving inconsistencies, aligning cost categories across provinces and districts, and ensuring the final dataset reflected the most reliable and comparable expenditure data possible. The microcosting analysis was then finalized by excluding Papua and Jakarta, resulting in data from 4 provinces and 4 districts, which allowed for a more precise and consistent comparison of expenditures. 2. To address potential biases arising from reliance on expert judgment due to data limitations, the study employed data triangulation by integrating multiple sources, including interviews, official reports, and program documentation. This process helped validate the expenditure data and strengthened the robustness of the findings. Furthermore, normalization was achieved by aligning cost categories and standardizing expenditure components across provinces and districts, ensuring greater consistency and comparability. These efforts enhanced the reliability of the microcosting analysis despite the inherent data gaps and variations across regions. 3. We acknowledge that the generalizability of the findings to regions with different health systems and socio-economic conditions is limited. However, the study's focus on diverse regions within Indonesia, each with distinct socio-economic profiles and local policies, provides valuable insights. By capturing the regional differences in health system challenges and strengths, the study highlights how these variations can inform policy development and health system improvement in regions with similar characteristics. In particular, the examination of multiple provinces with varying resources and local contexts allows for a deeper understanding of how specific challenges can be addressed and how successful strategies from one region may be adapted or improved for other areas. Furthermore, despite different characteristics, similar barriers and challenges were drawn and found across the regions. This comparative approach contributes to broader lessons on health system management, potentially benefiting other regions with similar contexts or challenges. | Lines 149 to 161:  *Expenditure data were collected from selected provinces and districts using a specialised format, modified from the existing DHO format, to address regional variations in financial reporting. Additional data were gathered through in-depth interviews with key informants from health offices and a review of program documentation for validation. Despite the structured format and interviews, some health offices could not provide detailed expenditure information, creating challenges in comparing quantitative data. To provide the robustness of the findings, data triangulation was employed, integrating interviews, official reports, and program documentation. Normalization was achieved by aligning cost categories and standardizing expenditure components across provinces and districts to ensure greater consistency and comparability. In cases of scarce or inconsistent data, these measures enabled a more reliable comparison of expenditures. The microcosting analysis was finalized by excluding Papua and Jakarta due to insufficient data, resulting in a final dataset from four provinces and four districts, allowing for a more accurate and consistent comparison of expenditures.*  Lines 614 to 618:  *While the study offers valuable insights specific to Indonesia, its generalizability to regions with different health systems and socio-economic conditions is limited. Nonetheless, by examining diverse regions within Indonesia with varying socio-economic profiles and policies, it highlights unique challenges and strengths that inform broader health system understanding and policy development in similar contexts.* |

**Comments from Reviewer 4**

|  | **Reviewer Comment** | **Response** | **Changes Made in Manuscript** |
| --- | --- | --- | --- |
| 1 | This is an excellent paper that documents and adds to the body of knowledge on an important lesson from the Covid pandemic. The paper is well written and presents clear lessons for future pandemic preparedness. This will be vital for the global health community and should be shared widely. Some minor final edits as outlined below would be beneficial. | We sincerely thank the reviewer for the generous and encouraging feedback. We are grateful for your recognition of the paper’s contribution to the field and its relevance to global health and future pandemic preparedness. We have carefully addressed the suggested minor edits and incorporated them into the revised manuscript. | - |
| 2 | Methods:  The authors could provide further explanation for choosing the 4 PH and 4 DHOs. Were they purposively chosen because of their proximity to the capital for instance, what criteria was used for choosing a particular province out of all the provinces that met a particular criteria? | Thank you for this comment. We appreciate the opportunity to clarify our province selection process.  The selection was not based on proximity to the capital but rather on the feasibility of data collection and the aim to reflect diverse regional contexts across the country. Areas with varying COVID-19 primary vaccination coverage (<50%, 50-70%, >70%), geographic challenges (such as remote, rural, urban, and island settings) were chosen to ensure that our findings capture a broad range of experiences. As a result, the final selection of PHOs and DHOs reflects a mix of regions and contexts, facilitating a comprehensive and comparative analysis of COVID-19 vaccine financial flows.  We have revised the study population section to include this explanation. | Lines 122 to 134:  *Among the provinces that met the vaccination coverage criteria, specific provinces were purposively selected based on the feasibility of data collection and geographic diversity. The selected provinces also represent different islands of Indonesia, supporting a more comprehensive understanding of regional variation. Within each selected province, one district was purposively chosen to capture intra-provincial variation and gather diverse perspectives from District Health Offices (DHOs). District selection considered not only vaccination coverage but also demographic and geographic characteristics. For instance, Sigi, Seram Bagian Barat, and parts of Tanggamus face accessibility challenges due to remote and mountainous terrains, while Gianyar represents a more urbanized district with stronger healthcare infrastructure. These varied contexts provided a solid foundation for comparing COVID-19 vaccine financial flows across regions. Initially, two provinces and two districts were selected for each vaccination coverage category; however, Jakarta and Papua were later excluded due to incomplete expenditure data.* |
| 3 | In the quantitative description, kindly explain how the expert judgement method was applied, to enable reproducibility. | Thank you for your comment. We have clarified the application of the expert judgement method in the quantitative description. In accordance with microcosting principles (Drummond et al. 2015), expert judgement was used to estimate the percentage use of resources such as supplies, personnel, equipment, transport, and other for each service component. Estimates were provided by experts with operational knowledge of the service delivery process. Data were collected using a structured questionnaire to ensure consistency and reproducibility. | Lines 164 to 168:  *Due to the unavailability of some specific data, we employed an expert judgement approach to estimate actual expenditure. Experts were asked to estimate the percentage use of resources including supplies, personnel, equipment, transport, and other relevant inputs for each service component. These estimates were provided by individuals with operational knowledge of the service delivery process.* |
| 4 | Results/Discussion:   a. Were any **reasons explored** or found for the difference in spending between the provinces? For instance; Tanggamus and Gianyar. b. In the limitation, kindly discuss a**ny possible impact of exclusion of the two provinces** without data on the study. | a. Yes, our study explored several factors contributing to differences in COVID-19 vaccination spending across provinces and districts. As discussed in the manuscript, these variations were shaped by fiscal capacity, government priorities, institutional and administrative capacity, geographic barriers, and private sector engagement. For example, Gianyar’s spending reflected strong collaboration with private and community stakeholders because it is a prominent tourist destination, reallocating broader COVID-19 response funds despite lacking a dedicated vaccination budget. As a green zone, it partnered with hospitals and private actors to support vaccination efforts. In contrast, Tanggamus, neither a national priority area nor a tourist destination, allocated more from its local budget to operational costs such as honorariums, transportation, and meals for vaccination teams. b. Thank you for the valuable suggestion. We have included a discussion in the limitation section addressing the possible impact of excluding Jakarta and Papua. Papua was excluded due to the lack of detailed expenditure data, as only total amounts without component breakdowns were available. Jakarta was excluded because its decentralized administrative system disperses budget realization data across multiple sub-district offices, preventing consolidation into a unified provincial dataset and making comparison with other provinces unfeasible. The exclusion of Papua may reduce representation of provinces with significant geographical challenges, but similar conditions are covered by including Maluku and Central Sulawesi. Although excluding Jakarta omits data from a province with high financing capacity and good regional access, Lampung provides comparable representation of financial capacity, and Bali represents similar ease of access within the study. | Lines 520 to 527:  *A comparison of Gianyar and Tanggamus highlights how local context shapes vaccination spending. Gianyar,as a big tourist destination and national priority area, received strong support from private sector and community partnerships, reallocating broader COVID-19 funds to support vaccination therefore no dedicated local budget. In contrast, Tanggamus, not being a national priority area, had to allocate and use primarily its own budget to cover COVID-19 vaccination program. These differences reflect variations in local capacity, national priority, governance, and stakeholder engagement influenced the prioritization and use of resources during the pandemic response.*  Lines 626 to 631:  *Additionally, we excluded Jakarta and Papua due to data limitations. The exclusion of Papua may limit representation of provinces with significant geographical challenges, but similar contexts are captured through Maluku and Central Sulawesi. Although excluding Jakarta omits data from a province with high financing capacity and good regional access, Bali and Lampung serve as comparable proxies for financial capacity and ease of access within the study.* |
| 5 | Conclusion  There are many important lessons from this study which are major for better pandemic preparedness, and the authors have highlighted these. The authors could further strengthen this section by reflecting further on **how other countries can apply these lessons in their own settings.** | Thank you for the input, here we add about lesson learn to use reallocation and consider the budget for reaching remote area population, especially for countries which want to build strategic for vaccine programme in remote area | Line 643 to 648:  *This study has broader relevance and can be a reference for the LMICs setting which had limited vaccine cold chain infrastructure and diverse archipelagic geography which faced similar challenges for decentralized vaccine programme delivery, especially during the emergency situation. Variations in budget commitment and spending can be minimised by having specific guidelines that prioritize core activities while allowing budget flexibility and funding mechanisms that can be mobilized to strengthen local governments capacity.* |
