# Supplementary material for: Tracking the financial flows of Indonesia’s COVID-19 vaccination program
Source: PLOS Glob Public Health. 2025 Aug 5;5(8):e0005041. doi: 10.1371/journal.pgph.0005041 (PMC12324125; doi:10.1371/journal.pgph.0005041)
Supplement: Attachment — Submitted filename: Response_to_Reviewers_auresp_2.docx [file pgph.0005041.s011.docx]

**Response to Reviewer**

Dear Editor,

We appreciate you and the reviewers for your precious time in reviewing our paper and providing valuable comments. It was your valuable and insightful comments that led to possible improvements in the current version. The authors have carefully considered the comments and tried our best to address every one of them. We hope the manuscript after careful revisions meet your high standards. The authors welcome further constructive comments if any.

Below we provide the point-by-point responses. All revisions can be found in the tracked changes version as well as in the clean version of the manuscript.

Sincerely,

Ayudina Larasanti

Senior Public Health Research Officer

**Center for Indonesia's Strategic Development Initiatives (CISDI)**

Jl. Probolinggo No. 40C, Menteng, Jakarta Pusat 10350

**Comments to the Author**

1. If the authors have adequately addressed your comments raised in a previous round of review and you feel that this manuscript is now acceptable for publication, you may indicate that here to bypass the “Comments to the Author” section, enter your conflict of interest statement in the “Confidential to Editor” section, and submit your "Accept" recommendation.

Reviewer #3: All comments have been addressed

Reviewer #4: All comments have been addressed

2. Does this manuscript meet PLOS Global Public Health’s [publication criteria](https://track.editorialmanager.com/CL0/https:%2F%2Fjournals.plos.org%2Fglobalpublichealth%2Fs%2Fcriteria-for-publication/1/010f0197a0dd493e-4af65158-6afa-4270-a7c1-98960eefd308-000000/OfXsFqFugJnyQ-GR2jfNmasDzAHx54rYASddwINQTG0=216)? Is the manuscript technically sound, and do the data support the conclusions? The manuscript must describe methodologically and ethically rigorous research with conclusions that are appropriately drawn based on the data presented.

Reviewer #3: Partly

Reviewer #4: Yes

3. Has the statistical analysis been performed appropriately and rigorously?

Reviewer #3: No

Reviewer #4: N/A

4. Have the authors made all data underlying the findings in their manuscript fully available (please refer to the Data Availability Statement at the start of the manuscript PDF file)?

The [PLOS Data policy](https://track.editorialmanager.com/CL0/https:%2F%2Fjournals.plos.org%2Fglobalpublichealth%2Fs%2Fdata-availability/1/010f0197a0dd493e-4af65158-6afa-4270-a7c1-98960eefd308-000000/_Mretjp9iLfQXbO6q4ecN_TyfFmxkw8Bdzui6znrNk0=216) requires authors to make all data underlying the findings described in their manuscript fully available without restriction, with rare exception. The data should be provided as part of the manuscript or its supporting information, or deposited to a public repository. For example, in addition to summary statistics, the data points behind means, medians and variance measures should be available. If there are restrictions on publicly sharing data—e.g. participant privacy or use of data from a third party—those must be specified.

Reviewer #3: Yes

Reviewer #4: Yes

5. Is the manuscript presented in an intelligible fashion and written in standard English?

PLOS Global Public Health does not copyedit accepted manuscripts, so the language in submitted articles must be clear, correct, and unambiguous. Any typographical or grammatical errors should be corrected at revision, so please note any specific errors here.

Reviewer #3: Yes

Reviewer #4: Yes

**Comments from Reviewer 3**

|  | **Reviewer Comment** | **Response** | **Changes Made in Manuscript** |
| --- | --- | --- | --- |
| 1 | The study offers critical insights into subnational COVID-19 vaccination financing in Indonesia over 2021 and 2022. However, it currently applies a fixed 2021 exchange rate (US$1 = IDR 14,308) to all expenditure data, without adjusting for exchange rate changes or domestic inflation between the two years. Given that vaccines and other key program inputs are predominantly imported, exchange rate depreciation and rising inflation in 2022 likely had a substantial impact on procurement costs and purchasing power at the subnational level. This could affect the accuracy of per capita expenditure comparisons and the interpretation of funding adequacy over time. The authors are encouraged to: (1) acknowledge this limitation in both the Methods and Discussion sections; and (2) where feasible, re-calculate or conduct a sensitivity analysis using the average 2022 exchange rate and incorporate inflationary/exchange rate adjustments to better reflect real-term expenditure changes | We converted the original figures from Rupiah to US dollars to facilitate international understanding and enable cross-country comparisons. In addition to this, a constant exchange rate was applied across the two-year analysis period to ensure consistency and avoid distortions caused by varying exchange rates. This approach, we believe, provides a more stable basis for comparing financing performance over time.  Since the transactions related to the vaccination program were conducted entirely in Rupiah, we deemed the use of different exchange rates for 2021 and 2022 unnecessary. Introducing annual exchange rate variations would shift the analytical focus away from the core objective: examining how the vaccination program was implemented across remote regions in Indonesia. Such an adjustment would introduce additional variables, requiring a separate analysis, as changing in exchange rate is influenced by many variables that lead to another analysis and different discussion that should be provided.  We also presented summary statistics on the exchange rate between mid-2021 and mid-2022 to further support our argument that fluctuations during this period were minimal. This reinforces the rationale for using a constant exchange rate, ensuring consistency without introducing undue variability into the analysis.  (1 USD to Rupiah)  Mean 14,423.88  Std Error 7.16  Median 14,419.74  Mode 14,419.74  Std Deviation 112.13  Sample Var 12,574.11  Kurtosis 0.82  Skewness 0.67  Range 654.26  Minimum 14,150.40  Maximum 14,804.66  source: (<https://www.bi.go.id/en/statistik/informasi-kurs/transaksi-bi/Default.aspx>)  Some studies using constant exchange rate can be found in prior studies, see Siregar et al. (2018) (<https://pmc.ncbi.nlm.nih.gov/articles/PMC6593591/>)  Regarding inflation, the Statistical Yearbook of Indonesia (BPS–Statistics Indonesia) reports that inflation for the health group made up 1.68% in 2021 and 2.87% in 2022 (<https://www.bps.go.id/en/publication/2023/02/28/18018f9896f09f03580a614b/statistik-indonesia-2023.html>). While inflation could be considered in cost calculations, we believe it has minimal influence on the outcomes presented in our study.  We appreciate the suggestion and acknowledge that incorporating exchange rate fluctuations and inflation adjustments might be more appropriate for a different type of analysis, such as evaluating the cost-effectiveness of imported vaccine components or estimating travel costs as a result of rising inflation. In such cases, substantial shifts in exchange rates could significantly impact inflation and, in turn, program outcomes, which is beyond the intended objectives of this study. | Lines 161-169 (Method Section-Quantitative)  *All expenditures were converted into US dollars using the 2021 exchange rate, with US$ 1 was equivalent to IDR 14,308. This conversion was conducted to enhance international comprehensibility and enable cross-country comparisons. A constant exchange rate was applied throughout the two-year analysis period to ensure methodological consistency and to avoid distortions arising from exchange rate fluctuations. This approach provides a more stable and comparable basis for assessing financial performance over time. Official statistics indicate that health-related inflation was relatively modest (1.68% in 2021 and 2.87% in 2022), indicating a minimal impact on the financial results presented in this study, and thus, no further inflation adjustment was deemed necessary.* |
